# Feed-Forward Segmentation of Figure-Ground and Assignment of Border-Ownership

**DOI:** 10.1371/journal.pone.0010705

**Published:** 2010-05-19

**Authors:** Hans Supèr, August Romeo, Matthias Keil

**Affiliations:** 1 Department of Basic Psychology, University of Barcelona (UB), Barcelona, Barcelona, Spain; 2 Institute for Brain, Cognition and Behavior (IR3C), Barcelona, Barcelona, Spain; 3 Catalan Institution for Research and Advanced Studies (ICREA), Barcelona, Barcelona, Spain; Mount Sinai School of Medicine, United States of America

## Abstract

Figure-ground is the segmentation of visual information into objects and their surrounding backgrounds. Two main processes herein are boundary assignment and surface segregation, which rely on the integration of global scene information. Recurrent processing either by intrinsic horizontal connections that connect surrounding neurons or by feedback projections from higher visual areas provide such information, and are considered to be the neural substrate for figure-ground segmentation. On the contrary, a role of feedforward projections in figure-ground segmentation is unknown. To have a better understanding of a role of feedforward connections in figure-ground organization, we constructed a feedforward spiking model using a biologically plausible neuron model. By means of surround inhibition our simple 3-layered model performs figure-ground segmentation and one-sided border-ownership coding. We propose that the visual system uses feed forward suppression for figure-ground segmentation and border-ownership assignment.

## Introduction

**Figure 1:**
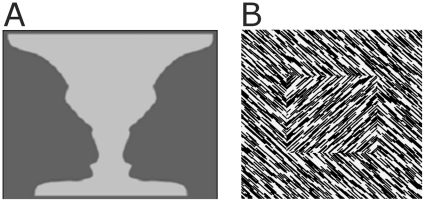
Examples of a Rubin vase (A) and a textured figure overlying a background (B). **A**: Bi-stable percept of a flower vase or two monkey faces depending on whether the borders between the luminance regions are assigned to the lighter or to the darker regions. **B**: The small centre square segregates from the background on basis of a difference in orientation of the line segments, and is perceived as a figure.

Figure-ground segmentation is achieved by assigning visual elements to either objects or background as a primary step in visual perception. Two main processes in organizing figure-ground segmentation are boundary assignment and surface segregation (Fig. 1). Boundaries are detected based on local contrast of visual elements, and are assigned to the figural region and not to the surrounding background region. This assignment is called border-ownership. For example in figure 1a, when the visual system assigns the contrast borders to the light grey area a vase is perceived on a black background. If the same contrast borders belong to the black regions two monkey faces are perceived and the light grey area becomes background. Surface segregation is based on the comparison of locally identified visual features across space. The surface is segregated from background by grouping operations according to Gestalt principles where similar elements are grouped into coherent objects. For example in figure 1b, the individual orientated line segments in the centre are grouped together because they have the same orientation and they are segregated from the elements in the surrounding region as they differ in orientation. Consequently a textured figure overlying a homogeneous background is perceived. So a key factor for figure-ground organization is the combination of local with global scene information. In the visual cortex contextual influences on neuronal activity have been interpreted as the neural substrate of figure-ground perception [1].

Intrinsic horizontal connections that connect surrounding neurons convey information from beyond the classical receptive field and can provide contextual information of the target stimulus. However, it has been shown that contextual suppressive effects come from large regions (4–7mm), while the horizontal spread of axons is limited (up to 3.5–4.5 mm radius in V1 monkey). Together with the slow conductance velocities (typically 0.1–0.2 m/sec) of these fibers, these observations cast doubt on a role for horizontal connections in perceptual integration. Feedback projections from higher visual areas to lower areas are more suitable to provide the contextual information necessary for figure-ground segmentation. Feedback projections have high conductance velocity (∼3–10 m/sec), have large spread in V1, and influence surround mediated responses in V1. Finally, theoretical and most, if not all, computer models explain figure-ground segmentation by recurrent processing through horizontal and/or feedback connections.

Yet several arguments are inconsistent with a leading role of feedback projections in producing contextual effects and figure-ground segmentation. For instance, V2 is the main contributor of feedback to the primary visual cortex, though inactivation of V2 has no effect on centre-surround interactions of neurons in the primary visual cortex [2]. Surround effects are primarily suppressive but blockade of intra-cortical inhibition does not reduce significantly surround suppression [3]. Surround suppression is fast and may arrive even earlier than the feedforward triggered excitatory classical receptive fields response [4], [5]. This timing is inconsistent with contextual modulation by late feedback. Also surround suppression in the monkey LGN emerges too fast for an involvement from cortical feedback [6].

**Figure 2:**
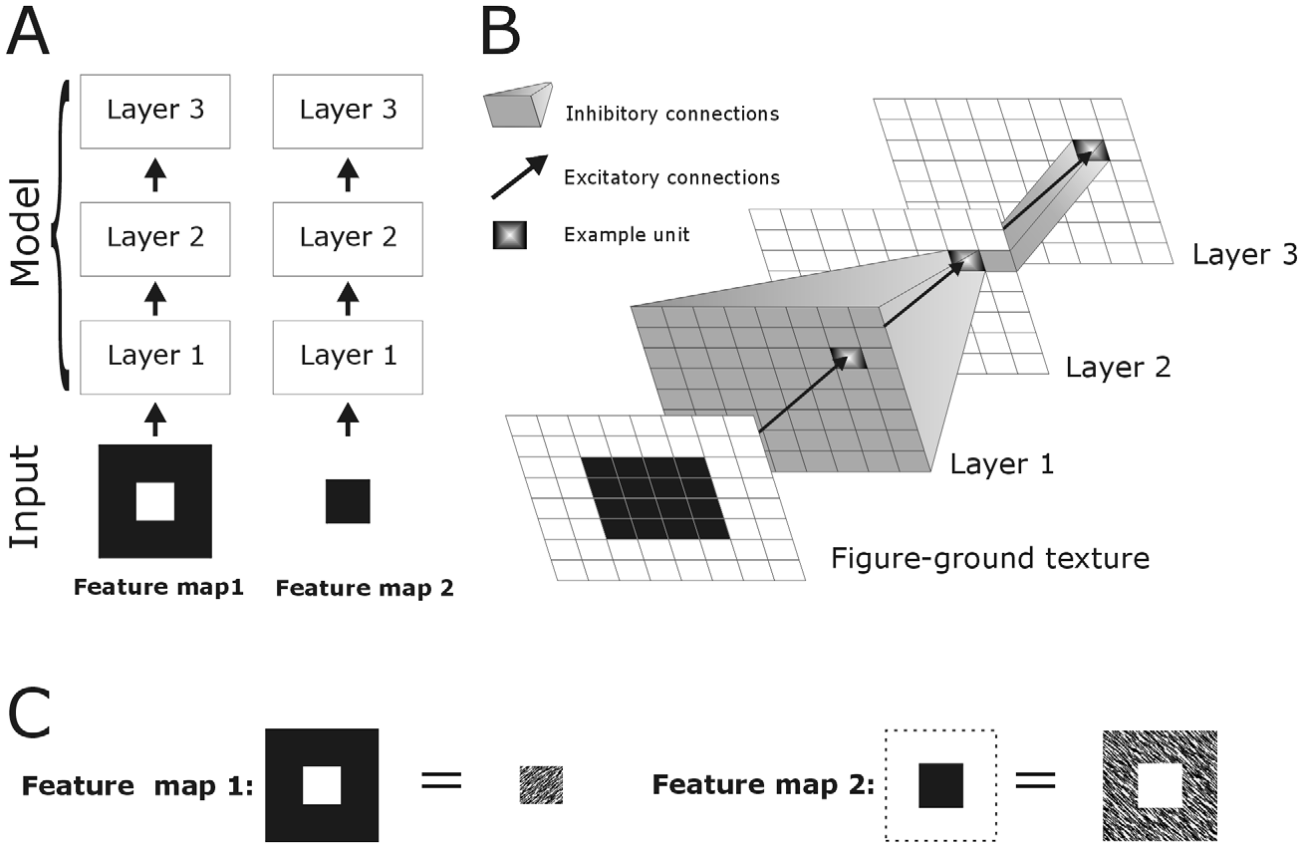
Architecture, connectivity and input scheme of the computational model. **A**: The model consists of three layers, which are unidirectional connected. Arrows define feedforward connections. The neural interactions are specific for feature preference. Lower two squares indicate the input (white regions) of the figure (left) and background (right). **B**: All layers receive point-to-point (retinotopic) excitatory input. Second and third layers also receive inhibitory input from all or one preceding neuron(s), respectively. **C**: The model input may correspond to the figure and background of a figure-ground texture as illustrated in figure 1b. Dotted lines demarcate the stimuli.

**Figure 3:**
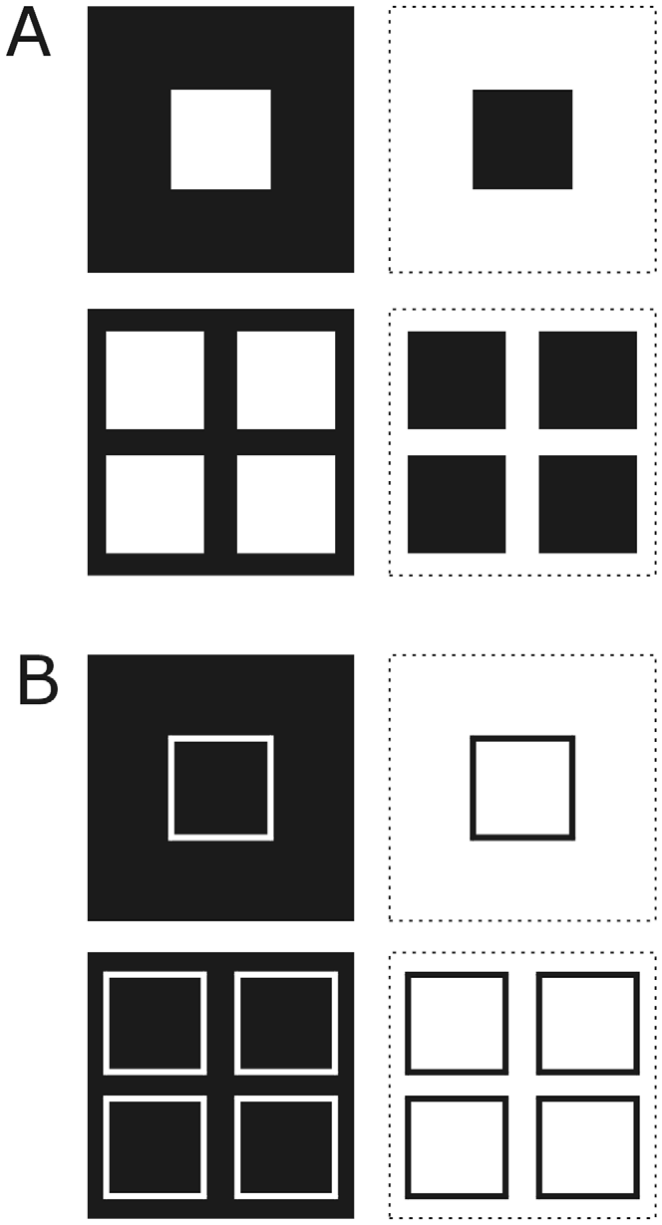
Figure-ground images of filled squares (A) and frames (B). **A,B**: White regions depict the input regions and black regions depict regions that provide no input to the feature specific neurons of the model. In the left column white squares represent the figures (A) and frames (B). In the right column the complementary shapes are illustrated where white regions represent the background. Dotted lines demarcate the stimuli.

**Figure 4:**
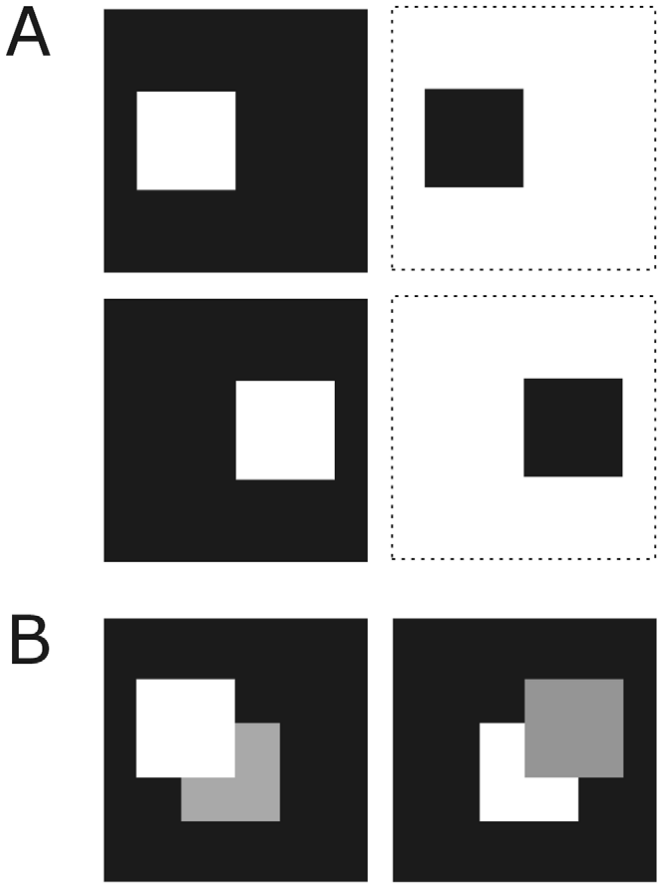
Figure-ground images. Figure-ground images of single squares (**A**) and two overlaying squares (**B**). Squares are shifted from the centre to illustrate one-sided border-ownership coding. Color coding is as in figure 3, except for the grey square which depicts an additional figure. Dotted lines demarcate the stimuli.

In contrasts, apart from carrying the sensory information, a role of feed-forward projections in producing surround effects related to figure-ground segmentation is unknown. Many findings, however, point out that contextual effects may modify the feedforward signal and extra-classical surround suppression is present at the first stages of sensory processing in the retina, LGN and V1. The aim of this study is therefore to have a better understanding of the role of feedforward connections in figure-ground organization, in particular in surface segmentation and border-ownership coding. For that reason we constructed a purely feedforward spiking model omitting horizontal and feedback connections (fig. 2) and tested the model for figure-ground segregation of textures (figs. 3,4) previously used in primate [7], [8] and computational [9], [10] studies. By means of feedforward surround inhibition, our simple 3-layered model performs figure-ground segmentation and one-sided border-ownership coding.

## Results

### Feature representation in layer 1

**Figure 5:**
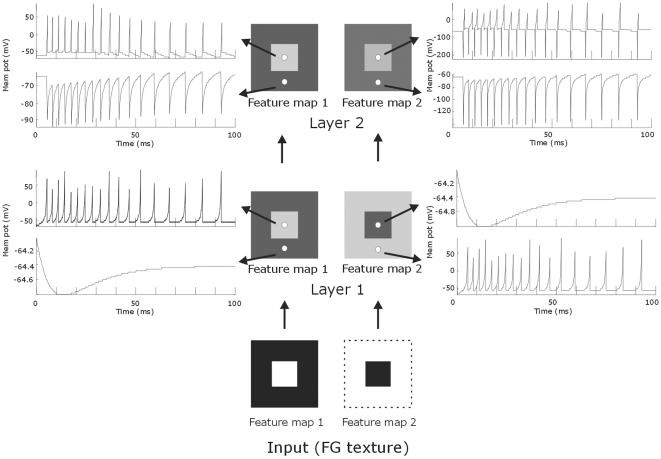
Model output and firing rates of neurons located on the figural region and on the background region. The light-dark squares in the centre column represent the NxN matrices of neurons of the model. The coloring of the matrices illustrates the membrane potential where light grey indicates high activity and dark grey zones low activity level. The white small circles depict neurons located on the figural and background regions. The arrows originating from them point to the corresponding spike responses of these neurons over time. Note that the activity pattern of the first layer of the model mirrors the texture input whereas the second layers only neurons at the figural region spike. Lower two BW squares represent the texture input, Dotted line demarcates the stimulus. Time is from stimulus onset.

The figure-ground image is accurately represented (fig. 5) because the input was mapped onto the first layer. So, only spiking neurons [11] at the figure location and the background regions, of the first and second feature map respectively were firing spikes. Initially these neurons had a higher firing rate (<100ms, 180 sp/s) and settle to a more constant firing rate (>100ms, 110 sp/s). Neurons that did not receive input from the figure-ground stimulus (black regions of the input patterns) showed a slight hyper-polarization before stabilizing the membrane potential around −64mV.

### Figure-ground segmentation in the second layer

**Figure 6:**
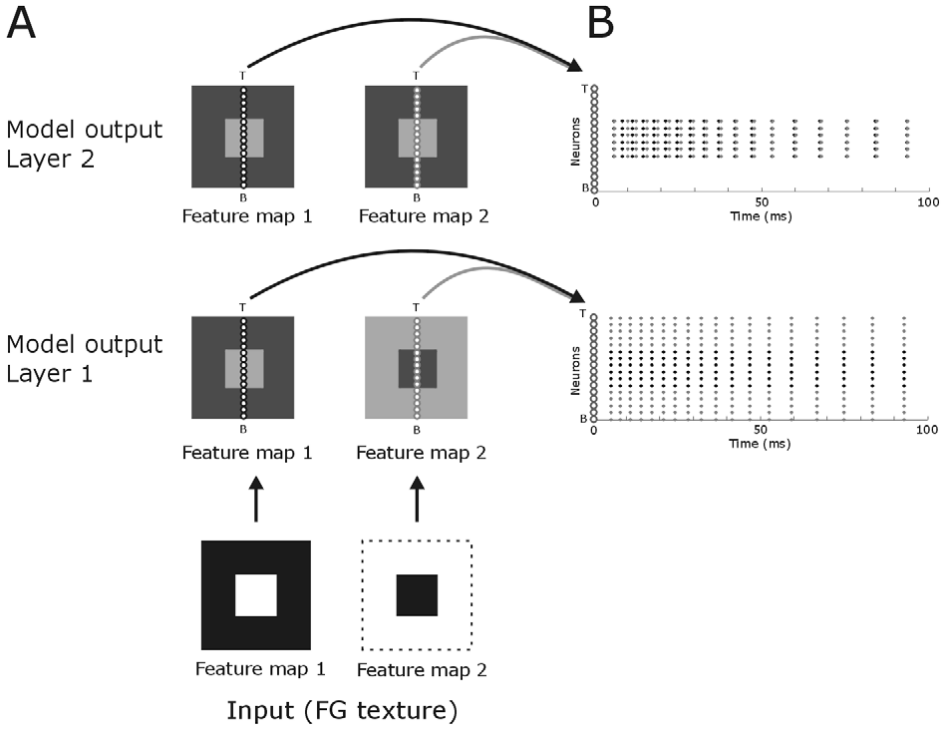
Distribution of spiking neurons after presenting the texture input to the model. **A**: The light-dark squares in the centre column represent the NxN matrices of neurons of the model. The coloring is as in figure 5. The lines of small white circles denote an entire column of neurons of an NxN matrix. We used N-16 to clearly illustrate the distribution of the spiking pattern. T and B signify top and bottom of the matrix, respectively. Arrows point to the spiking behavior of these neurons. Dotted line demarcates the stimulus. **B**: The neurons from (A) are here plotted on the *y*-axis (small, white circles). Each black and grey dot represents a spike from the corresponding neuron on the *y*-axis. Spikes from neurons from feature map 1 are in black and spikes from feature map 2 neurons in grey.

Whereas neurons in the first layer received continuous input from the figure-ground image, neurons in the second layer received spiking input from the first layer. Each neuron received retinotopic excitatory input and global inhibitory input from all spiking neurons in the first layer. For feature map 1 (the central figure) the spatial pattern of spiking activity in the second layer mirrored the excitatory input pattern (figs. 5,6). In contrast, for feature map 2 (background) the spatial activity pattern changed compared to the input pattern. Neurons that received excitatory input became quiescent and neurons that did not receive excitatory spiking input fired spikes. This result is explained by rebound spiking as a results of the relative strong global inhibitory input. So in the second feature map many layer 1 neurons were activated by the relatively large background region, which provoked a strong suppression of all layer 2 neurons. For the neurons located on the background this inhibition neutralized the retinotopic activation. For the neurons located at the centre (representing the figure location) this global inhibitory signal was the sole input resulting in a strong and rapid hyper-polarization of the membrane potential, which caused rebound spiking of these cells. Such a phenomenon of surround activation of otherwise un-stimulated neurons has also been described in primate V1 [12]. Moreover, our observation agrees with the notion of cue invariant figure-ground segregation in the visual cortex [13], [14]. Thus for both feature maps figure-ground segregation was achieved; neurons located in the central figural region were active while background neurons were salient. The activation of layer 2 neurons by global inhibitory input was thus independent of direct retinotopic sensory input. Note, however, that rebound spiking is *not* essential for segregating figure from ground; important here is that background neurons are silent.

### Assignment of border-ownership in the third layer

**Figure 7:**
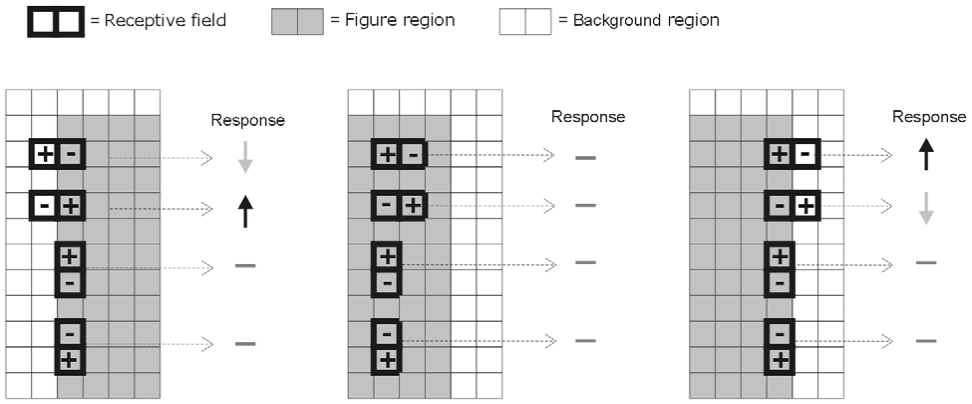
Illustrations of subfields of the receptive fields of layer 3 neurons. Each neuron receives an excitatory (+) and an inhibitory (−) input from neighboring layer 2 neurons. Depending on the combination of neighbors, 8 possible distributions of subfields are possible. Grey shading indicates part of the figural region and white regions indicate the background. One-sided border ownership can be achieved when layer 3 neurons receive excitatory input from a layer 2 neuron i.e. when it is located on the figure region, and inhibitory input from a neuron located on the background. In all other cases layer 3 neurons will be silent or inhibited. The bars/arrows next to each neuron (grey circles) indicate the response.

Besides figure-ground signals, many (18% in V1 and 59% in V2) neurons in early visual cortex show selective responses to contour borders [15]. In particular neurons in V2 preferably respond to the contour when it belongs to one side of a figural region and not to the other side of the figure; a phenomenon called one-sided border-ownership assignment. In order to explain one-sided border ownership, we applied a basic aggregation of separate sub-regions of receptive fields [16], [17] where neurons in the third layer receive both an excitatory and an inhibitory connection from two neighboring neurons located in the second layer of the same feature map. In this way, borders can be detected if the excitatory sub-region receives feedforward input and the inhibitory sub-region does not. For example, layer 3 neurons respond when the excitatory sub-region falls on the figural part and the inhibitory one falls on the background (fig. 7). In essence, the idea of opposite receptive field sub-regions is reminiscent of the opponent model for border-ownership coding as proposed by Zhou et al. [15].

**Figure 8:**
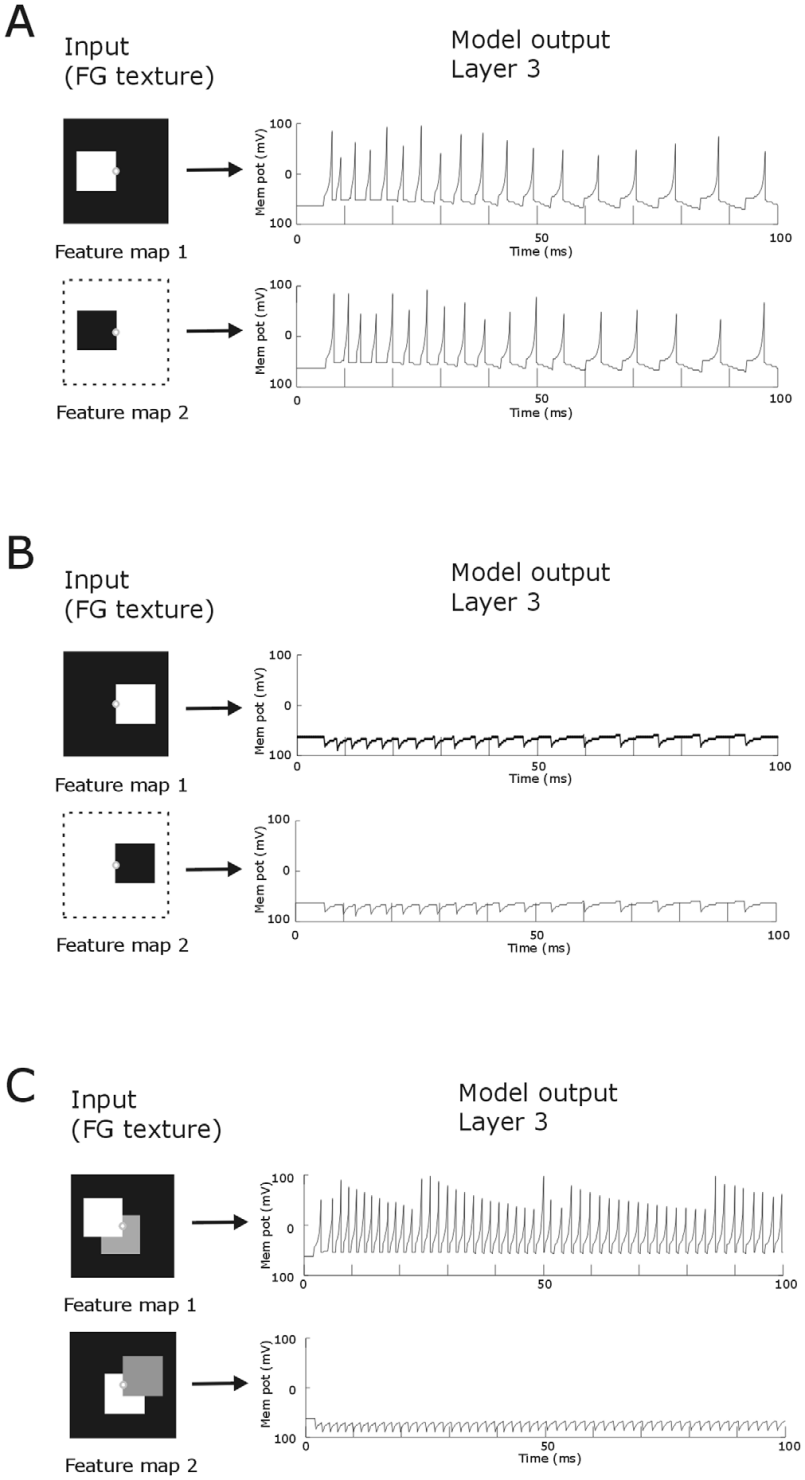
One-sided border-ownership assignment. **A**: A neuron spikes when the border of the figure is on the left side of the receptive field. **B**: When the border is on the right side of the receptive field, the neuron does not spike. **C**: One-sided border-ownership for two partially overlapping figures. The neuron spikes when the border belongs to the figure at the left and not when it belongs to the right figure. Note that the receptive field stimulations are identical in both conditions. Small white circles indicate the location of the receptive field of the neuron. Dotted line demarcates the stimulus. Time is from stimulus onset.

Figure 8 shows the border-ownership coding for a single figure and for two partially overlapping figures. Here neurons respond only when the border of the figure is located at the left side of the receptive field. Activation of both excitatory and inhibitory sub-regions will not lead to a neural spiking response in the third layer. So, the surface of the figure is not detected. In the case of two overlapping figures, the local contrast between the two figures should be sufficient large to determine border-ownership assignment.

There are 3 types of edge detection cells described: edge contrast polarity, border-ownership and a combination of them. In principle our model can reproduce all the different types of neurons that signal contrast borders by applying different combinations of sub-regions from the second layer and/or from the first layer and from the two feature maps. A further product of such combinations of sub-regions is that neurons coding border-ownership are orientation selective. This has also been described in the visual cortex where edge detection is mainly observed for neurons that have an orientation preference [15].

### Figure size, number and frames

**Figure 9:**
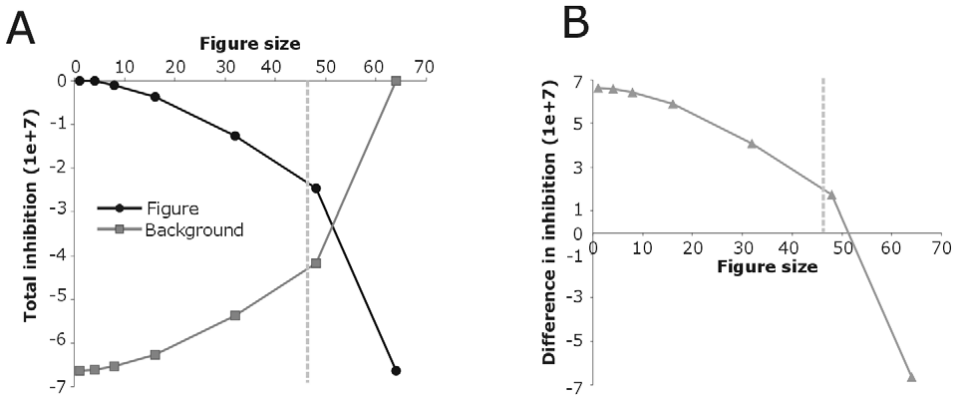
Total inhibitory input as a function of figure size. **A**: The total amount of inhibitory input that a neuron receives increases for larger figures for neurons located on the figure. For neurons located on the background, the total inhibition decreases with figure size. **B**: Here the difference between inhibition for neurons located on the figure and for neurons on the background is plotted. Vertical dotted lines indicate the maximal figure size that the model correctly segregates.

In the visual cortex, contextual interactions are complex and heterogeneous and are observed for stimuli far outside the classical receptive field. For textures stimuli, Zipser [13] reported figure-ground modulation in V1 for figures up to 10–12°. They further reported a dependency of modulation strength on the figure size. Size tuning of surround suppression has also been reported for drifting sinusoidal gratings up to 10 degrees in V1 and LGN [3]. Similarly, surround effects for uniform stimuli extend 20 degree up to 40 degrees beyond the classical receptive field [18]. In the case of border ownership, contextual effects are observed for stimuli 20° from the target stimulus and show only mildly size dependency [15]. To test the model behavior for stimulus size we applied different figure sizes. Figures as small as 1×1 pixels up to figures sizes of 46×46 pixels are detected properly. Compared to small figures large figures (>32×32 pixels) have a ∼40% weaker response modulation than the smaller figures (180 sp/s vs. 145 sp/s). The same is true for the border-ownership signal in the third layer as it is based on the occurrence of figure-ground signal in the second layer. These results can be explained by the fact that inhibition increases by enlarging the figure size thereby lowering the total input to the neurons (fig. 9). The inhibitory contribution to the input of layer 2 neurons as a function of figure size is shown in figure 9. Because the responses of layer 2 neurons to the change in inhibition do not follow the same rule, figure sizes (e.g. 46×46 pixels) larger than background size can still be detected correctly.

**Figure 10:**
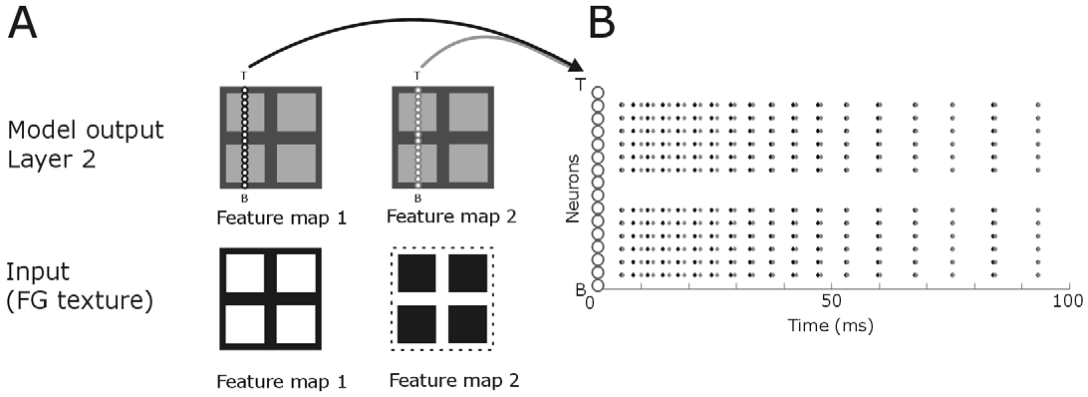
Distribution of spiking neurons after presenting four figures to the model. **A**: The lines of small white circles denote an entire column of neurons of an NxN matrix. We used N-16 to clearly illustrate the distribution of the spiking of neurons. Arrows point to the spiking pattern. The light-dark squares in the centre column represent the NxN matrices of neurons of the model. The coloring is as in figure 5. Dotted line demarcates the stimulus. **B**: The neurons from (A) are here plotted on the *y*-axis (small, white circles). Each black and grey dot represents a spike from the corresponding neuron on the *y*-axis. Spikes from neurons from feature map 1 are in black and spikes from feature map 2 neurons in grey.

**Figure 11:**
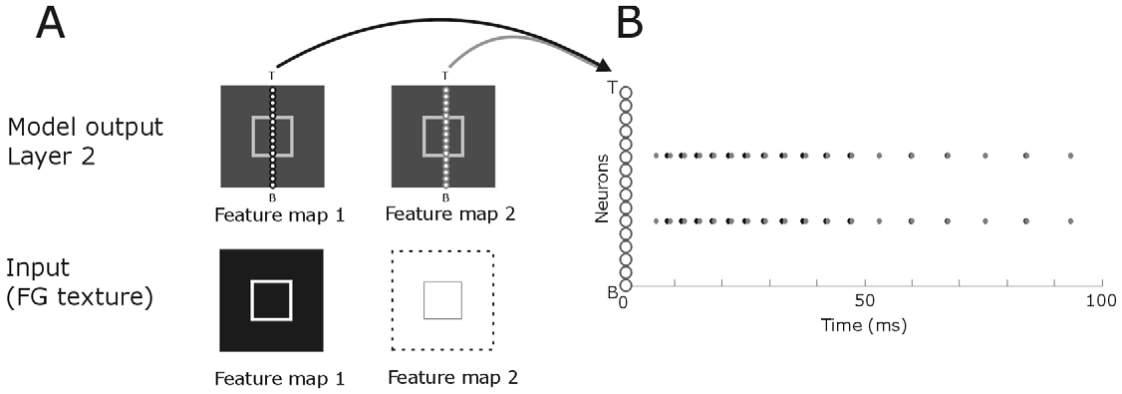
Distribution of spiking neurons after presenting an outline to the model. **A**: The lines of small white circles denote an entire column of neurons of the NxN matrix of the model from the different layers. We used N-16 to clearly illustrate the distribution of the spiking pattern. Arrows point to the spiking behavior of these neurons. Coding is as in figure 5. Dotted line demarcates the stimulus. **B**: The neurons from (A) are here plotted on the *y*-axis (small, white circles). Each black and grey dot represents a spike from the corresponding neuron on the *y*-axis. Spikes from neurons from feature map 1 are in black and spikes from feature map 2 neurons in grey.

**Figure 12:**
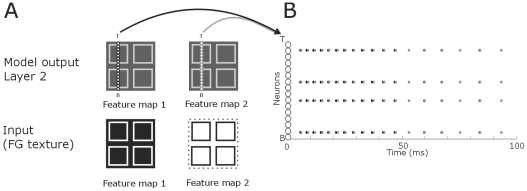
Distribution of spiking neurons after presenting four outlines to the model. **A**: The lines of small white circles denote an entire column of neurons of the NxN matrix of the model from the different layers. We used N-16 to clearly illustrate the distribution of the spiking pattern. Arrows point to the spiking behavior of these neurons. Coding is as in figure 5. Dotted line demarcates the stimulus. **B**: The neurons from (A) are here plotted on the *y*-axis (small, white circles). Each black and grey dot represents a spike from the corresponding neuron on the *y*-axis. Spikes from neurons from feature map 1 are in black and spikes from feature map 2 neurons in grey.

We also tested the model for more than one figure. In figure 10 the results of figure-ground segregation are presented for four figures. Earlier psychophysical experiments demonstrate that these textures are not ambiguous, i.e. the multiple squares are perceived as figures and not as background [19]. For multiple figures, feature detection occurs in the first layer while figure-ground segregation is observed in the second layer and border-ownership in the final layer. We also tested the network performance for the outline of one or multiple figures. Outlines were 1 pixel wide containing concave and convex regions. The inner part of the outline was part of the second feature map, i.e. part of the background. The results show that for both feature maps, the outline is detected accurately for a single figure as well as for multiple figures (figs. 11,12).

### Figure-ground contrast

**Figure 13:**
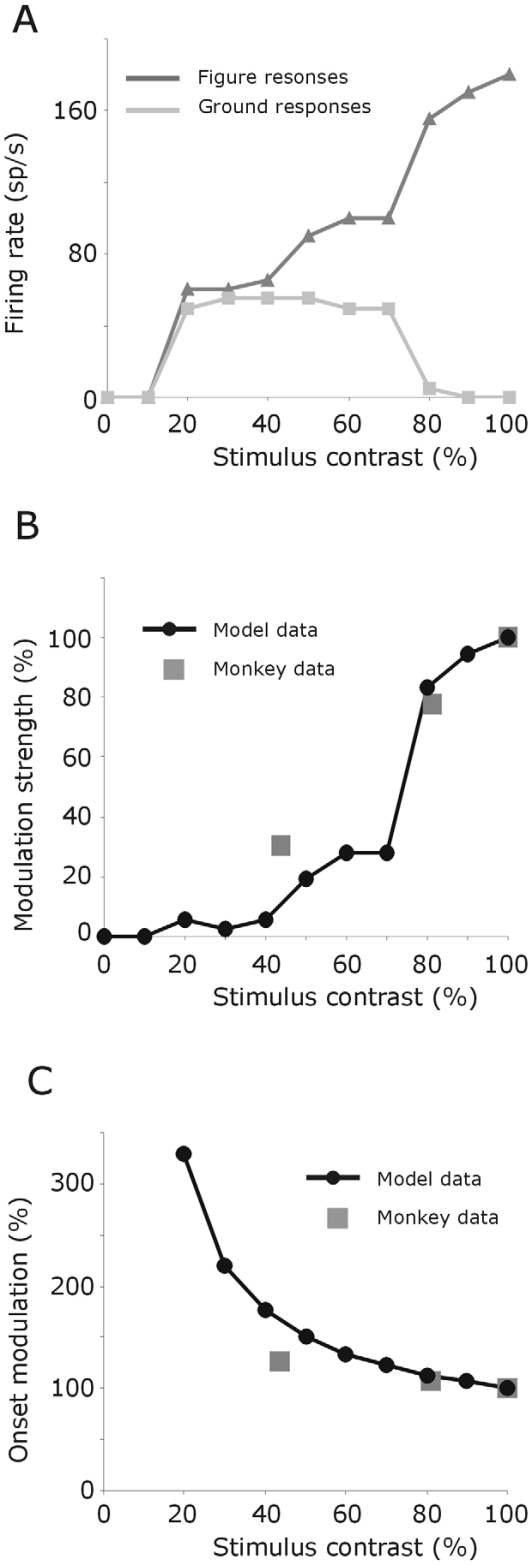
Responses to figure and ground as a function of stimulus contrast. **A**: Average firing rate to figure and ground. **B**: Modulation strength (figure minus ground responses). Grey squares represent the modulation strength for different figure-ground textures observed in visual cortex of monkeys. **C**: Onset latencies of figure-ground modulation. Grey squares represent onset latencies for different figure-ground textures observed in monkey visual cortex. The high contrast stimulus used by Supèr (Supèr et al., 2001) is set here to 100% for comparison.

We tested the model for different figure-ground contrasts. To do so, we decreased the input pixels values from 1 to 0, in steps of 0.1. Lowering the figure-ground contrast causes a gradual weakening of figure-ground signal; an effect produced by weaker figure responses and higher background responses (fig. 13a,b). Such a push-pull operation also takes place during figure-ground segregation in the monkey visual cortex ([8]; see fig. 13b). Here, compared to responses to homogeneous textures, responses to figure elements are enhanced and responses to ground elements, where a figure is presented outside the receptive field, are weakened. This push-pull effect becomes less by lowering the stimulus contrast [8]. Besides stronger response modulations, our data show that increasing contrast produces a shorter onset latency of the figure-ground signal (fig. 13c). For a high contrast figure, the onset latency is about 3 times shorter compared to a low contrast figure; a phenomenon also observed in the visual system ([20]; see fig. 13c). Similar results hold true for border-owner assignment because in our model the occurrence of border-ownership assignment is directly related to the timing of figure-ground segregation.

## Discussion

The goal of the present study was to examine the role of feedforward connections in figure-ground operations. We found that our three layered model of spiking neurons could perform figure-ground segregation and one-sided border-ownership assignment in a purely feedforward manner. The feedforward segregation of figure from ground was robust. A decrease of the input contrast by 80% still yielded figure-ground segregation. Figure-ground segregation occurred for very small figures (even for the size of 1×1 pixel) and for large figures. Since the surround inhibition depended on stimulus size, figure-ground segregation failed when the figure size approximated the background size. This agrees with human figure-ground perception, where small stimuli are interpreted as figures and larger ones as background. When figure and background have the same size the assignment of figure and ground becomes ambiguous (see e.g. [21]).

### Figure-ground segregation & one-sided border-ownership assignment

**Figure 14:**
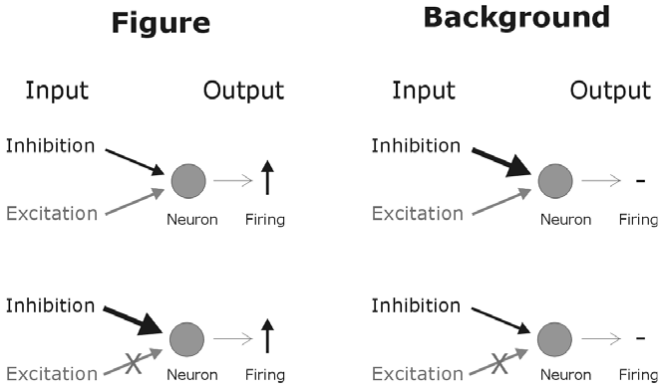
Scheme illustrating the mechanisms of figure-ground segregation by the model. A neuron located on the figure region (left panels) receives weak global inhibitory input together with retinotopic excitatory input. As a result the neuron fires spikes. In the case when a neuron receives strong global inhibition and no excitation, rebound spiking occurs. Neurons on the background region (right panels) are silent. The strong global inhibitory input cancels the excitatory drive and weak inhibitory input does not produce rebound spiking.

The first layer transformed the figure-ground texture input into a spike map, which was send to the layer 2 neurons. In the second layer, neurons received retinotopic excitatory input and global inhibitory connections from all the spiking neurons in the preceding layer of the corresponding feature map. In the first feature map only a minority of the total number of neurons, those at the figure location, contributed to the global inhibitory effect. Consequently each neuron in the second layer received relatively weak inhibition, which was not sufficiently strong to cancel out the excitatory activation of the neurons at the figure location. Neurons outside the central figure region only received global inhibition and remained silent (fig. 14). In the other feature map however, the numerous layer 1 neurons receiving background input produced together a strong global inhibitory input to each second layer neuron. This inhibitory input was strong enough to cancel out the excitatory activation, thereby silencing the background neurons if the second layer. For those layer 2 neurons that did not receive excitatory input, i.e. the ones at the central figure location, the strong global inhibition resulted in rebound spiking (fig. 14). Thus, in the second feature map layer 2 neurons at the figure location fired and neurons at the background were silent. Hence, also here the model segregated figure from ground. Note that the important point for figure-ground segregation to take place is not the fact that the figure neurons of the second feature map spike but that the background neurons are silent (or fire few spikes). In the third layer, neurons received one excitatory and one inhibitory connection from two neighboring neurons in the second layer. Such a combination of spatially separated receptive field sub-regions reproduced one-sided border-ownership assignment (see fig. 8).

### Can figure-ground segmentation occur without feedback?

Feedback projections from higher visual areas to lower areas are believed to provide the contextual information necessary for figure-ground segmentation. Yet several studies indicate that feedback projections may not be the sole component for producing contextual effects and figure-ground segmentation. For instance, V2 is the main contributor of feedback to the primary visual cortex, though inactivation of V2 has no effect on centre-surround interactions of neurons in the primary visual cortex [2]. Surround effects are primarily suppressive but blockade of intra-cortical inhibition does not reduce significantly surround suppression [3]. Surround suppression is fast and may arrive even earlier than the feedforward triggered excitatory classical receptive fields response [4], [5]. This timing is inconsistent with contextual modulation by feedback. Also surround suppression in the monkey LGN emerges too fast for an involvement from cortical feedback [6].

Moreover, removing feedback (including V3, V4, MT, MST, but not V2) to V1 impairs figure-ground perception, but does not affect visual detection of textured figure-ground stimuli [22]. This finding implies that figure-ground segmentation occurs without feedback from these extra-striate areas, and without producing visual awareness. This agrees with the belief that figure-ground organization is an automatic process [23]. For example, preserved figure-ground segregation is observed in neglect patients [24] and surface segregation signals evolve independent of attention [19]. Similarly, the assignment of border-ownership precedes object recognition and the deployment of attention [23], [25]. Furthermore, the short onset latencies and sometimes incomplete cue invariance suggest that border-ownership assignment is not generated in higher level visual areas but within the lower visual areas [15]. In addition, figure-ground segmentation depends on the size of the figure region and drops with increasing figure sizes (>8°–12°). This size dependency argues against segregation by feedback since termination fields of feedback projections cover large regions of visual space in V1. Finally, an intriguing finding is that contextual neural interactions corresponding to perception are observed at sub-cortical levels in the LGN and even in the retina [26] and that competition for object awareness is fully resolved in monocular visual cortex [27]. So, there is considerable evidence against a unique role of feedback in figure-ground segregation and supports the idea for a feedforward component in figure-ground segmentation.

### Possible correspondence of the model architecture to the visual system

Visual information entering the retina produces graded potentials which are converted by ganglion cells into action potentials. Our model neurons in the first layer convert continuous texture input into spikes, and therefore the first layer can correspond to the ganglion cell layer of the retina. The second layer of the model may represent the LGN as the main recipient of ganglion connections. The retinotopic excitatory connections mimic the powerful synaptic excitatory contacts that each LGN neuron receives from one to three retinal ganglion cells [28]. The same retinal ganglion cells also provide inhibitory postsynaptic currents [29]. The influence of inhibition in the LGN however comes from a larger retinal region than that from excitation. Likely this is because retinal ganglion cell activate inter-neurons resulting in inhibition beyond those directly activated by ganglion cells [29]. This feedforward inhibition is fast; it takes place at the very beginning of an event related response [5], [29]. For instance, some types of IPSC faithfully follow the EPSC with a latency of 1 ms and they are tightly locked to visual stimulation [29]. In our model we reproduced the fast surround inhibition seen in the retinogeniculate system by combining in time the retinotopic excitatory and the global inhibitory input. If our second layer indeed corresponds to the LGN, then figure-ground segregation, particularly for contrast-defined figures, does not start in the cortex but already in the thalamus. Although the existence of figure-ground signals in the LGN are not known, contextual responses matching perception, and attention signals are described in the LGN (e.g. [26]).

Alternatively, the second layer of our model may correspond to V1. In this case, LGN present just a relay of retinal information. In a previous version we successfully tested this by adding an extra layer representing the LGN. The thalamocortical connections are highly convergent maintaining the retinotopic mapping in V1. In V1, they synchronously activate layer 4 spiny cells, which in turn activate directly the upper layer neurons. Furthermore, thalamocortical synapse specifically and strongly excite the fast spiking network [30]. Fast spiking neurons form an inhibitory network connected through electric synapses and mediate thalamocortical feedforward inhibition [31]. In the visual cortex feedforward inhibition can suppress large regions [3], [4], [31], [32] and is fast where it can arrive even earlier to the target neuron than excitatory signals [4]. Within the cortex, conductance of fast spiking interneuron onto spiny layer 4 neurons is ∼10 fold greater than that of excitatory conductance [32] and fast spiking cells mediate strong and fast (<∼6 ms) thalamocortical feedforward inhibition that can shunt thalamocortical excitation [31], [32]. Intra-cortical surround inhibition, on the other hand is rather slow, tens of milliseconds [33]. In our model the combination in time of excitatory and strong inhibitory inputs mimic the synchronous activation and the strong and fast feedforward inhibition described in the visual system. Finally, inhibition from the surround has been shown to be orientation or direction selective [34]–[38]. In our model, surround inhibition is also feature, e.g. orientation, specific.

The last layer may represent V2, which receives its main feedforward input from V1. Neurons in V2 aggregate V1 receptive fields at similar but not identical topographical locations. In such a design V2 neurons show spatial in-homogeneity in the two-dimensional receptive field structure. V2 receptive fields contain sub-regions that are tuned to similar or dissimilar orientations [16], [17]. Accordingly, the response properties of V2 neurons are principally determined by the distribution of the aggregation of V1 receptive fields. A further complexity added to V2 receptive fields is that V2 neurons combine both excitatory and inhibitory parts of separated receptive fields of V1 neurons [17]. Many of the interactions between the sub-regions are inhibitory, which might be of V2 intra-cortical origin or inherited from V1 [17]. By applying two antagonistic sub-regions of V2 receptive fields we reproduced a simple form of sub-field aggregation of V1 receptive fields. Such a design explains border-ownership assignment. Alternatively input to V2 may come from LGN cells, in particular the non-standard cells, which project directly to V2. This idea is supported by the observation of V1–V2 correlograms that are centered on zero indicating coincidence of firing by common input. However, the LGN-V2 connection and its functions are yet poorly described.

### Onset latencies of figure-ground signal and border-ownership coding

A notable outcome of our model is that the figure-ground signal pops-out immediately after receiving the first spikes, both at the border and at the centre of the figure. Also border-ownership assignment occurred at similar time as figure-ground segregation. At first glance this may seem odd compared to the often reported late onset of figure-ground segregation in the visual cortex. General, non-specific surround suppression is one of the earliest contextual effects, which takes about 7 ms to develop after response onset [39]. The orientation specific modulation of responses to centre-surround stimuli occurs a bit later, around 15–20ms after the response onset [39]. Lamme showed onset latencies for figure-ground modulation of 60–120 ms after stimulus onset, which equals to 30–60 ms after response onset [40]. In another study, early textured figure–ground segregation was seen to occur at 40–80 ms after stimulus onset [41] and was not different between V1 and V2 neurons. In this study, figure–ground segregation started 20–60 ms after the response onset. Border-ownership assignment for color and grey stimuli starts at ∼70 ms after stimulus onset, both in V1 and V2 [15]. This is within 10 ms in V1 and within 25 ms in V2 after response onset. Thus, although frequently described as having a late onset, neural signatures of figure-ground segregation and assignment of border-ownership can arise fast (as fast as 10–20 ms after response onset) both in V1 and V2. So at a closer look, our findings of 5 ms after stimulus onset agree with the fastest reported onset latencies of figure-ground signals. Moreover, in the visual system features, like orientation needs first to be computed before figure-ground segregation can take place. So the time that is needed to process features is included in the described onset latencies for the occurrence of figure-ground modulation. In our model however feature specificity was implicitly encoded and thus did not add extra time to the onset time of figure-ground segregation. So, when corrected for a latency of ∼10 ms for orientation tuning to take place, our figure-ground latencies are close to the observed ones in the visual system.

### Segregation of boundary and surface of the figure

Boundary detections and surface filling-in are other issues related to the onset of figure-ground segregation. Neurophysiological observations show that figure-ground modulation occurs first at the border of the figure followed by modulation for the center region of the textured figure [40]–[42]. These findings can be interpreted as a filling-in process or, alternatively, as two independent processes of border detection and a grouping operation where surface responses simple lag behind the responses to border. The finding that surface signals and not boundary signals are reduced by extra-striate lesions [40] argue for two distinct mechanisms. Also, the finding that the onset of the modulated responses across the whole surface is the same [40] argues against a gradual filling-in process of textured stimuli over time and favors independent mechanisms for boundary and surface detection. Our data shows that the whole figure popped-out instantaneously and no filling-in process of the figural region took place. Therefore, our model data fit the idea of two independent mechanisms for local border and surface detection. Local border detection, however, is absent in our model. The absence of border detection is explained by the fact that border detection is based on the comparison of local features, where discontinuities form a boundary. To detect local discontinuities interactions between features are needed. In our model such interactions were not implemented and thus boundary detection is not possible.

### Other models on figure-ground segregation

Many computational models exists explaining figure-ground segregation and border-ownership. Most, if not all, computer models [9], [10], [43]–[57] explain figure-ground segmentation by recurrent processing through horizontal and/or feedback connections, as suggested in the neurophysiological literature.

One model [58] may appear to be feed forward. However, their conductance based model does not use DEQs. Therefore, it can easily be re-defined and interpreted as a feedback model. More importantly is that the Sakai & Nishimura 2006 model is based on surround fields (iso-orientation suppression and cross orientation facilitation). These surround fields were not explicitly modeled but numerous (hundreds) different positions and sizes of surround fields were designed and tested. The neural origin of these surround effect are based on the information within V1 [35], [36]. It has been demonstrated that in the visual system surround effects are mediated by long range horizontal connections [59]. In fact some models rely on lateral connections for figure ground organization [49] and thus agree with the Sakai & Nishimura 2006 model. Thus on the first sight the model of Sakai & Nishimura 2006 may give the impression of a pure feed forward connectivity scheme. However, taking into account the surround fields, the Sakai & Nishimura 2006 model implicitly includes lateral connections.

Most of the models are conductance based models excluding the rich and complex response behavior of neurons. Some models rely on lateral connections for figure ground organization [49] and demonstrate that feedback is in principle not necessary. However, lateral latencies in the visual cortex are too long to explain contextual effects in figure-ground organization. Other studies add feedback projections to improve the performance of the model. The role of feedback is to suppress noise and to enhance figure-ground effects [48], [54], [55]. These results fit the idea that top down control has a push-pull effect where relevant signals are enhanced and irrelevant signals suppressed.

### Limitations and predictions of our model

Our intention was to test feedforward segregation of textures that previously had been studied in primates and computer models, and which are believed to depend on recurrent processing. The model was not designed for complex or natural images, neither was the intention to obtain state of the art figure-ground segmentation. On the contrary, to understand the role of feedforward connections in figure-ground segregation we constructed a minimalistic feedforward architecture. Therefore, we deliberately omitted recurrent processing, thereby severely constraining the possible outcomes of the model. For example, feature interactions are not possible with the current network because the lack of horizontal connections. Nevertheless, our simple network advocates a feedforward organization of figure-ground. According to our model data, one-sided border-ownership coding does not depend on local feature contrast but is based on surface segregation of the figure. Thus, our model predicts that local border detection and border ownership coding employ different neural mechanisms. Furthermore, in our model we modeled global inhibition by adding a negative weight to the feedforward connections and not by introducing local inhibitory cells at layer 2. In this way the combination in time of excitatory and strong inhibitory inputs mimic the synchronous activation and the strong and global inhibition described in the early visual system. Further studies should reveal how figure-ground segregation occurs by including inhibitory cells. Finally, considering the simplicity of our model figure-ground segregation may occur already at the earliest stages of visual processing.

### Conclusion

In the visual system it is not possible to separate axonal circuits and to analyze their function in isolation. Computational modeling of neural networks offers a complementary role to allow dissecting axonal circuits. Using biophysical realistic spiking neurons, we tested to what extent feed-forward connections contribute to the neural mechanisms underlying figure-ground organization. Our simple, 3 layered feed-forward spiking model performs figure-ground segmentation and one-sided border-ownership coding. It turns out that global inhibition and rebound spiking are important ingredients for figure-ground organization. We conclude that figure-ground organization includes besides feedback also a feedforward component.

## Methods

### Model architecture

The model is composed of three layers, each containing two arrays of N×N units or neurons of the Izhikevich type ([11]; see fig. 2). For all layers, we used N = 64. Lower and higher values of N were also tested and did not affect model performance. The two separate arrays of each layer represent two neuronal cell populations with opposite preference for a single feature.

### Connections

The feedforward connections between the layers are divided into excitatory and inhibitory connections (fig. 2b). All excitatory connections are retinotopic (point-to-point connections) where neuron N*_ij_* in one layer solely connects to neuron N*_ij_* in the next layer. Thus the excitatory part of a neuron's receptive field has size one. The pattern of inhibitory connections differs between layers. Neurons in the first layer do not receive inhibitory signals from the texture input. In the second layer all neurons of a feature map (see below *Inputs*) receive inhibition from all neurons located in the same feature map of the first layer. In the third layer, a neuron N*_ij_* receives feature specific inhibition from a neighbor of neuron N*_ij_* located in the second layer. In principle, there are eight neighbors; for simplicity we chose only one (see fig. 2b). Inhibition is achieved by assigning negative weights to the connections. Neither intra-laminar connections, i.e. horizontal connections between neurons within or across feature maps, nor feedback connections, i.e. connections from higher layers to lower layers, are included in the network architecture.

### Inputs

The studied textured figures are arrays of N×N pixels, with N as in the model, containing one or four centered squares (fig. 2c,3a). Input arrays are binary (0 or 1) and correspond to the preference of a single visual feature, like luminance, orientation, direction of motion, color etc. In other words, 1 stands for optimal tuning whereas 0 is the opposite. For every shape its binary complementary is also included (fig. 2c,3a). The complementary input thus represents the reverse preference of the visual feature. These two arrays are referred to as feature map 1 and feature map 2. For instance, the first one corresponds to the orientation of line segments in the centre square of figure 1b and the second to the surrounding line segments, which have the opposite orientation (see fig. 2c). Together they form the figure-ground texture. The two feature maps are processed by separated neuronal pathways (channels). We also used the outlines of the figures as input (fig.3b). For border-ownership coding single squares were placed to either the left or the right side for clarifying the side preference (fig. 4a). Also two partially overlapping squares were used for border-ownership (fig. 4b). In this case, the two small squares (figures) belong to one feature map. The pixel values of the additional square were 0.3.

### Neuronal cell type

Hodgkin–Huxley models are too slow for network operation and integrate-and-fire models are unrealistically simple and incapable of producing rich spiking and bursting dynamics exhibited by cortical neurons. We opted to use the spiking neurons of Izhikevich [11]. These neurons combine the biologically plausibility of Hodgkin–Huxley-type dynamics and the computational efficiency of integrate-and-fire neurons, and are capable of producing rich firing patterns exhibited by real biological neurons. We choose the neurons to be phasic bursting because feedforward connections rely on bursting neurons, which report the beginning of the stimulation by transmitting a burst. In the brain bursts are important to overcome the synaptic transmission failure and reduce neuronal noise. Also they can transmit saliency of the input and bursts can be used for selective communication between neurons.

### Model dynamics

Cell dynamics is described by the ‘simple’ spiking model of Izhikevich (1, 2)
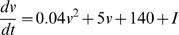
(1)

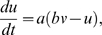
supplemented with the after-spike reset rule
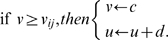
(2)
*v*, *u*, *I*, *t* are dimensionless versions of membrane voltage, recovery variable, current intensity and time. Further, *a* is a time scale, *b* measures the recovery sensitivity, *c* is the reset value for 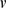, and *d* is the height of the reset jump for 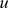. For all our simulations *a* = 0.02, *b* = 0.25, *c* = −55, *d* = 0.05, and 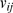 = 30. These values correspond to the phasic bursting type of the Izhikevich neuron [11]. In the evolution law for V (eq. 1), a capacitance factor C was omitted [11]. When dimensions are reintroduced, voltages are read in mV and time in ms. As initial conditions at *t_0_* = 0 we set
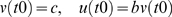
for all the positions in our arrays (since we deal with two-dimensional objects, equations (1) and (2) are actually meant for 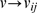, 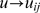, 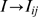, *i,j* = 1, …, N, and condition (3) is in fact applied to 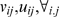. We used the Euler method with 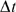 = 0.20 ms. The input current *I* in (1) is the result of summing different matrix contributions of the form
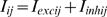
(4)where ‘exc’ stands for ‘excitatory’, ‘inh’ for ‘inhibitory’, and i,j are spatial indices. Further, for layers 1 and 2,
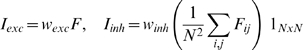
(5)
*F* is either the two dimensional figure itself or the binary array defined by the presence of spikes, i.e., with ones where condition (2) is satisfied and zeros elsewhere. The 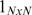 symbol denotes an NxN matrix containing just ones. Since excitatory receptive fields have size one, excitatory signals are point-by-point (retinotopic) copies of *F* itself, multiplied by the corresponding weight. The inhibitory part, whose associate receptive field has the same size as *F*, produces a spatially constant term –hence the 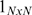 matrix- which is proportional to the normalized sum of all the *F* coefficients times the inhibitory weight. Thus, all layer 2 units within a feature map received the same inhibitory input. In our design, the employed weights are 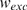 = 3, 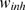 = 0 for the texture input and 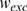 = 400, 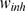 = −900 for the signals from layer 1 to layer 2. The weights values are a result of a heuristic process, and can be changed without critically affecting the model performance.

The path from layer 2 to layer 3, where border-ownership assignments take place, may be described in terms of two receptive sub fields, inhibitory and excitatory, both of size one and next to each other. Their working is more easily expressed by means of the convolution
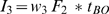
(6)

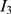
 indicates the total input to layer 3, 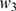 is the weight (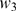 = 200), 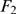 means the spike map at layer 2, and the applied filter is given by the 2×1 matrix 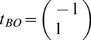.
